# Post-traumatic Isolated Superior Rectus Hematoma Treated With Oral Steroids

**DOI:** 10.7759/cureus.81763

**Published:** 2025-04-05

**Authors:** Abdul Hadi Mallick, Zubaida Sirang

**Affiliations:** 1 Ophthalmology and Visual Sciences, Aga Khan University Hospital, Karachi, PAK

**Keywords:** corticosteroid treatment, diplopia, hypotropia, orbit, superior rectus muscle

## Abstract

We present a rare case of an isolated right superior rectus hematoma in a 14-year-old boy following a road traffic accident (RTA), which was effectively treated with oral corticosteroids alone. This case emphasizes the importance of prompt diagnosis and conservative treatment in isolated extraocular muscle hematomas. It also highlights the role of clinical awareness and imaging in guiding treatment while avoiding unnecessary surgery. Additionally, the case raises awareness about the rare presentations of orbital trauma and the effectiveness of non-invasive treatments.

## Introduction

Isolated extraocular muscle hematomas are uncommon injuries, often resulting from blunt trauma to the orbit. The superior rectus muscle is rarely affected alone due to its protected position within the orbit, shielded by surrounding structures. Accurate diagnosis is crucial to avoid complications like diplopia, restriction of gaze, or optic nerve damage. Delayed recognition can lead to long-term visual deficits and functional impairments. Clinicians need to understand the mechanisms, symptoms, and management of these injuries. To the best of our knowledge, there have been only three reported cases in the English medical literature of superior rectus hematomas following blunt trauma [1-3], so documenting them is important to improve awareness and patient outcomes in similar cases.

## Case presentation

A 14-year-old boy presented to the emergency department after his car collided with a tree on the 19th of December 2024. He was in the passenger's seat at the back but without his seatbelt. His head likely struck the right side of the door, and he lost consciousness, having no memory of the incident. He was initially kept in the ICU until he regained consciousness and then stepped down to the general ward. At the presentation, there were multiple facial abrasions and lacerations, including a right eyebrow and lip laceration, which were sutured. There was also right periorbital swelling, which decreased during his stay. He was discharged on the 23rd of December 2024. After discharge, he noticed double vision and drooping of the right upper lid even after periorbital swelling had subsided, so with these complaints, he presented to us on the 1st of January 2025.

**Figure 1:**
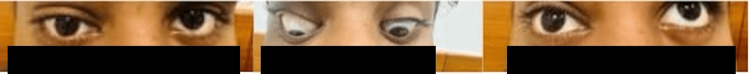
Clinical appearance of extraocular movements at presentation. Right eye hypotropia, upward gaze limitation, and ptosis.

His primary complaint was double vision. His visual acuity was 20/30 in the right eye and 20/25 in the left eye, with the best corrected visual acuity being 20/20 in both eyes. On further examination, there was significant hypotropia, with around 3 mm ptosis, mild proptosis, and diplopia in all gazes (Figure 1).

**Figure 2:**
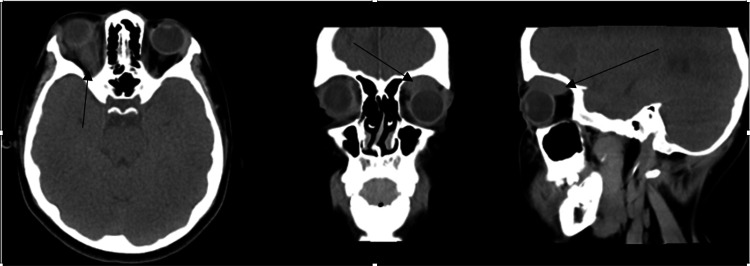
Axial, coronal, and sagittal views of CT scan image showing fullness in the right superior rectus muscle, likely hematoma (black arrow).

There was no reactive afferent pupillary defect (RAPD) in either eye, and pupils were round, regular, and reactive. Slit lamp and fundus examinations were well within normal limits. Suspecting an orbital floor fracture with entrapment of the inferior rectus and/or inferior oblique or a "trap door fracture," we ordered a computed tomography (CT) scan and asked the patient to follow up the next day with the reports. The CT scan showed an intact orbital floor but an enlarged superior rectus muscle, likely representing a superior rectus hematoma. All other extraocular muscles (EOMs) were normal and intact. There was no evidence of any other ocular pathology on CT (Figure 2). 

**Figure 3:**
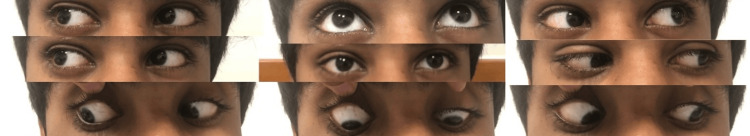
EOMs are full in all gazes with no ptosis or proptosis. EOMs: extraocular movements

Treatment was started with oral prednisolone 40 mg/day with slow tapering doses for three weeks, and the patient was instructed to return if symptoms do not improve or exacerbate. He followed up on the 21st of January 2025 with a complete resolution of diplopia, ptosis, and proptosis. EOMs were full in all gazes (Figure 3). Both orthoptic examinations and Hess charting were well within normal limits. His visual acuity (VA) was also 20/20 in both eyes using the standard Snellen chart.

## Discussion

Superior rectus hematomas are rare and may resemble other orbital conditions like orbital cellulitis, muscle entrapment, or retrobulbar hemorrhage. Blunt trauma can lead to vascular injury in the muscle, causing localized bleeding [1-3]. The superior rectus muscle is protected by the bony orbit, making isolated hematomas unusual. These cases often present with nonspecific symptoms, which can make diagnosis challenging. Clinicians must maintain a high level of suspicion when a patient with orbital trauma presents with diplopia and restricted gaze.

One of the most common causes of post-traumatic diplopia is a blowout fracture with entrapment of either the inferior rectus or inferior oblique or both in the floor of the orbit [3], and, in our case, it was also one of the top differentials until the CT scan showed a normal orbital floor and only isolated fullness of the superior rectus muscle.

There are numerous similarities when we compare our case to the previous three reported cases in the literature [1-3]. The mechanism of injury is blunt trauma in all of the cases with comparable clinical and radiological findings. The management option opted for by all three cases was also conservative, using oral corticosteroids. On follow-up, complete recovery was observed in diplopia and ptosis.

In this age group, a 'white-eyed' fracture is also suspected. It is a subgroup for which urgent repair is required to avoid permanent neuromuscular damage. The scenario is generally seen in patients less than 18 years of age, typically with little visible external soft tissue injury, and usually affects the orbital floor. It involves the acute incarceration of herniated tissue in a 'trapdoor' effect and occurs because of the greater elasticity of bone in younger people. Patients may experience acute nausea, vomiting, headache, and persistent activation of the oculocardiac reflex can occur [3].

Other differential diagnoses include retrobulbar hemorrhage and muscle entrapment, particularly in the context of trauma. The forced duction test can help differentiate between mechanical entrapment and neurogenic causes [4-6]. CT imaging is the best diagnostic tool for identifying hematomas, fractures, and other injuries. MRI can help evaluate soft tissue when dealing with chronic or evolving lesions [7].

In most cases, treatment is conservative, involving observation and corticosteroids to reduce inflammation and promote hematoma resolution [8-10]. Surgery is typically reserved for cases with optic nerve compression, persistent hematomas, or orbital compartment syndrome [11]. In rare cases, persistent diplopia may require strabismus surgery or other interventions [12]. Early corticosteroid treatment, as shown in this case, can significantly reduce recovery time and prevent long-term complications.

This case demonstrates a rare cause of diplopia, hypotropia, and ptosis following blunt trauma to the orbit with complete resolution following conservative management with oral corticosteroids. The favorable outcome suggests that non-surgical treatment is effective in uncomplicated cases if there is no optic nerve involvement or worsening symptoms. It also highlights the importance of collaboration between ophthalmology, radiology, and emergency medicine to ensure optimal patient care.

## Conclusions

Isolated superior rectus hematomas are uncommon; however, they should be considered in individuals who have diplopia and orbital injuries. When there is no acute orbital compartment syndrome or injury to the optic nerve, conservative treatment with oral steroids can produce great results. For successful results without surgery, early detection, suitable imaging, and careful follow-up are essential. The significance of thorough clinical examination and the possibility of positive results with non-invasive treatment approaches are both emphasized in this case.
